# Slow-wave sleep, oxygen desaturation, and memory consolidation in sleep-disturbed individuals

**DOI:** 10.1016/j.ijchp.2025.100574

**Published:** 2025-05-06

**Authors:** Yi-Chun Kuan, Hsin-Wei Lin, Cheng-Chang Yang, Jung-Lung Hsu, Wen-Te Liu, Chaur-Jong Hu, Arnab Majumdar, Yi-Chih Lin, Chih-Wei Peng, Cheng-Yu Tsai

**Affiliations:** a Sleep Center, Taipei Medical University-Shuang Ho Hospital, New Taipei City 235041, Taiwan; b Department of Neurology, Taipei Medical University-Shuang Ho Hospital, New Taipei City 235041, Taiwan; c Department of Neurology, School of Medicine, College of Medicine, Taipei Medical University, Taipei 110301, Taiwan; d Taipei Neuroscience Institute, Taipei Medical University, Taipei 110301, Taiwan; e Department of Biomedical Engineering, National Taiwan University, Taipei 100233, Taiwan; f School of Medicine, College of Medicine, Kaohsiung Medical University, Kaohsiung 807378, Taiwan; g Department of Clinical Psychology, College of Medicine, Fu Jen Catholic University, New Taipei City 242062, Taiwan; h Department of Neurology, New Taipei Municipal TuCheng Hospital, New Taipei City, Taiwan; i Department of Neurology, Chang Gung Memorial Hospital Linkou Medical Center, Neuroscience Research Center, and College of Medicine, Chang-Gung University, Taoyuan, Taiwan; j Division of Pulmonary Medicine, Department of Internal Medicine, Taipei Medical University-Shuang Ho Hospital, New Taipei City 235041, Taiwan; k Research Center of Artificial Intelligence in Medicine, Taipei Medical University, Taipei 110301, Taiwan; l School of Respiratory Therapy, College of Medicine, Taipei Medical University, Taipei 110301, Taiwan; m Department of Civil and Environmental Engineering, Imperial College London, London, UK; n Department of Otolaryngology, Taipei Medical University-Shuang Ho Hospital, New Taipei City 235041, Taiwan; o Department of Otolaryngology, School of Medicine, College of Medicine, Taipei Medical University, Taipei 110301, Taiwan; p School of Biomedical Engineering, College of Biomedical Engineering, Taipei Medical University, Taipei 110301, Taiwan; q Research Center of Sleep Medicine, College of Medicine, Taipei Medical University, Taipei 110301, Taiwan; r Research Center of Thoracic Medicine, Taipei Medical University, Taipei 110301, Taiwan

**Keywords:** Obstructive sleep apnea, Polysomnography, Slow-wave sleep, Memory consolidation

## Abstract

Slow-wave sleep (SWS) plays a crucial role in memory consolidation, yet its disruption in obstructive sleep apnea (OSA) remains poorly understood. This study investigates the relationship between SWS characteristics, nocturnal oxygen desaturation, and memory performance in individuals with sleep disturbances. This cross-sectional study included 49 participants with memory complaints and sleep disturbances who underwent overnight polysomnography (PSG) and cognitive assessments to determine the presence and severity of OSA. SWS parameters, including the slow-wave index, amplitude, and duration, were extracted from PSG data alongside the apnea-hypopnea index (AHI) and oxygen desaturation index (ODI-3 %). Memory consolidation was assessed pre- and post-sleep using the Word Sequence Learning Test (WSLT), with the WSLT-Memory Index Score (WSLT-MIS) as the primary outcome measure. Multiple linear regression models adjusted for age, BMI, and education were used to analyze associations between sleep parameters and memory outcomes. Higher ODI-3 % and AHI were significantly associated with poorer memory consolidation, as indicated by lower WSLT-MIS scores (p < 0.05). AHI during NREM sleep was more strongly associated with poorer memory consolidation compared to AHI during REM sleep. Conversely, a higher slow-wave index was positively correlated with better WSLT-MIS scores and retention rates (p < 0.05). These findings highlight the critical role of SWS in memory consolidation and the detrimental effects of OSA-related sleep disturbances. While CPAP therapy remains the standard treatment for improving oxygenation and reducing sleep fragmentation in OSA, additional strategies aimed at enhancing SWS may further support cognitive function. Longitudinal studies and neuroimaging approaches are needed to better understand the mechanisms linking SWS enhancement and cognitive health.

## Introduction

Specific types of memory, such as episodic, procedural, and working memory have been shown to be particularly susceptible to sleep-related disruptions (Twigg et al., 2010; Vanek et al., 2020). A study on older adults found that worsening memory was associated with the presence of new or persistent sleep disturbances, whereas improved memory correlated with better sleep quality (Wills et al., 2022). Sleep is a fundamental biological process that plays a crucial role in cognitive health, particularly in facilitating memory consolidation (Paller et al., 2021). Among the various sleep stages, slow-wave sleep (SWS) is essential for stabilizing and integrating newly acquired information into long-term memory. This process is mediated by synchronized neocortical slow oscillations, hippocampal sharp-wave ripples, and thalamocortical spindles, which facilitate the transfer of information from the hippocampus to the neocortex (Born, 2010). Additionally, SWS supports brain health through its involvement in the glymphatic system, which aids in clearing metabolic waste products such as amyloid-β, a protein implicated in Alzheimer’s disease (AD) (Astara et al., 2023; Balkrishnan et al., 2023).

However, disruptions in sleep architecture, commonly observed in conditions such as obstructive sleep apnea (OSA), can impair these processes. OSA is characterized by intermittent hypoxia, sleep fragmentation, and reductions in SWS and rapid eye movement (REM) sleep. These disturbances have been linked to cognitive deficits across various domains including attention, executive function, and memory (Bubu et al., 2020; Villagran & Scullin, 2019). In particular, episodic and procedural memory appear to be most vulnerable to the effects of OSA (Ahuja et al., 2018; Bucks et al., 2013; Twigg et al., 2010). Despite these established associations, the precise mechanisms through which OSA-related sleep disturbances contribute to cognitive impairments remain incompletely understood (Ercolano et al., 2024).

Memory processing involves three primary stages: encoding, consolidation, and retrieval (Melton, 1963). Among these, SWS plays a critical role in memory consolidation by enhancing synaptic plasticity and neural synchronization. During SWS, the reduced levels of acetylcholine facilitate hippocampal-neocortical communication, which is integral to stabilizing newly encoded memories (Born, 2010). However, disruptions in SWS reduce the frequency and amplitude of slow-wave oscillations, impairing this critical process (Cellini, 2017; Rosenzweig et al., 2015). Moreover, such SWS disruption can hinder glymphatic clearance of neurotoxic proteins, potentially accelerating neurodegenerative processes (Astara et al., 2023).

Intermittent hypoxia further exacerbates these effects by inducing oxidative stress and neuroinflammation (Snyder et al., 2017). These physiological changes may lead to hippocampal damage and synaptic plasticity alterations, thereby impairing both memory consolidation and retrieval (Kloepfer et al., 2009; Squire et al., 2015). Studies have shown that individuals with OSA exhibit poorer performance on procedural memory tasks and verbal retention tests compared to healthy controls (Kloepfer et al., 2009; Verghese et al., 2022). These findings underscore the dual impact of SWS disruption and intermittent hypoxia on cognitive function.

Prior research has examined the distinct cognitive effects of sleep fragmentation, daytime sleepiness, and hypoxemia, all of which often co-occur in individuals with OSA but may differentially affect cognitive domains. Sleep fragmentation has been shown to primarily impair sustained attention, vigilance, and executive functioning, likely due to disrupted sleep continuity and impaired prefrontal cortical activity (Lal et al., 2022; Seda & Han, 2020). Daytime sleepiness is associated with deficits in vigilance and psychomotor slowing, yet it does not consistently correlate with the severity of OSA, indicating that different underlying mechanisms are involved (Seda & Han, 2020). Hypoxemia, defined by intermittent oxygen desaturation, has been more specifically linked to hippocampal-dependent memory impairments, possibly mediated by oxidative stress and neuroinflammation processes (Lal et al., 2022; Seda & Han, 2020). PStudies utilizing comprehensive neuropsychological batteries and experimental paradigms, such as working memory tasks, procedural memory tests, and attentional control measures, have successfully differentiated the cognitive contributions of these OSA-related mechanisms (Lal et al., 2022; Lau, 2023; Seda & Han, 2020). Incorporating these nuanced distinctions into future research may help clarify the domain-specific cognitive consequences attributable to each pathophysiological feature of OSA.

The cognitive consequences of OSA extend beyond immediate memory impairments to an increased long-term risk of neurodegenerative diseases such as AD (Bubu et al., 2020; Ercolano et al., 2024). The mechanisms underlying these impairments likely involve alterations in sleep architecture, heightened sleep fragmentation, and disturbances in both SWS and REM sleep (Cellini, 2017; Rosenzweig et al., 2015). However, the interactions between nocturnal oxygen desaturation, SWS disruption, and cognitive decline remain poorly characterized (Thorisdottir et al., 2024). Furthermore, traditional neuropsychological assessments may not sufficiently detect subtle cognitive deficits linked to chronic sleep disturbances, highlighting the need for specialized diagnostic tools tailored to this population (Olaithe et al., 2021).

To address these gaps, this study investigates the relationship between nocturnal oxygen desaturation, SWS disruption, and memory consolidation in individuals experiencing sleep disturbance. By analyzing polysomnography (PSG) data alongside pre- and post-sleep cognitive performance measures, this research seeks to elucidate the mechanisms by which sleep disturbances impair memory processes. We hypothesize that greater nocturnal oxygen desaturation and reduced SWS activity will be associated with poorer memory retention, particularly in tasks requiring consolidation during sleep. The findings have the potential to inform clinical strategies aimed at improving both sleep quality and cognitive function in populations affected by OSA.

## Methods

### Participants

This cross-sectional study recruited participants presenting with memory problems and sleep disturbances from a sleep center at a university-affiliated hospital between July 2020 and January 2021. PSG data and cognitive assessment outcomes were collected for all participants. The inclusion criteria were: (1) 35-80 years of age, (2) at least elementary-level education, and (3) normal or corrected-to-normal visual acuity. The exclusion criteria included a history of major neuropsychiatric disorders, such as dementia, Parkinsonism, epilepsy, stroke, severe head trauma, schizophrenia, bipolar disorders, or major depressive disorder. Participants who did not meet inclusion criteria or had incomplete data were excluded from the final analysis. The diagnosis of OSA was confirmed through overnight PSG, conducted as part of the study protocol. This study received approval from the Taipei Medical University–Joint Institutional Review Board (TMU–JIRB: N202004048), and all procedures adhered to ethical guidelines, including data collection, de-identification, and statistical analyses.

### PSG data collection

In-laboratory overnight PSG was conducted using the Embla N7000 (ResMed, San Diego, CA) or Embletta MPR system (Natus Medical, Pleasanton, CA) at the sleep center of Shuang-Ho Hospital, a medical center affiliated with Taipei Medical University. Sleep staging and respiratory event scoring were performed using RemLogic software (version 3.41; Embla Systems, Thornton, CO). The following oximetry parameters were used to assess oxygenation status: apnea-hypopnea index (AHI), arousal index (ArI) and oxygen desaturation index (ODI-3 %). These indices were selected based on their established relevance in prior studies assessing the cognitive effects of sleep-related hypoxia (Tsai et al., 2022). The thresholds used for defining apnea, hypopnea, and desaturation episodes followed the 2017 American Academy of Sleep Medicine (AASM) guidelines, ensuring standardization with current clinical practice (Berry et al., 2017).

Apnea was defined as a ≥ 90 % reduction in oronasal thermal sensor signals, while hypopnea was identified by a ≥ 30 % decrease in nasal pressure sensor signals with ≥ 3 % desaturation or the occurrence of an arousal event. Desaturation episodes were characterized by a ≥ 3 % drop in mean oxygen saturation lasting for at least 10 seconds. Electroencephalography (EEG) recordings followed AASM guidelines, using frontal (F4), central (C4), and occipital (O2) electrodes referenced to the right mastoid (M1). The EEG sampling was set to 250 Hz with a band-pass filter of 0.3–35 Hz. For arousal scoring, an abrupt shift in brainwave signals was identified if alpha (8–12 Hz), theta (4–8 Hz), or high frequency (> 16 Hz, excluding spindle activity) was detected for at least three seconds, following at least ten seconds of stable sleep. OSA severity was classified as follows: none (AHI < 5 events/h), mild (AHI 5–15 events/h), moderate (AHI 15–30 events/h), and severe (AHI ≥ 30 events/h).

### Slow-wave detection

**Fig. 1:**
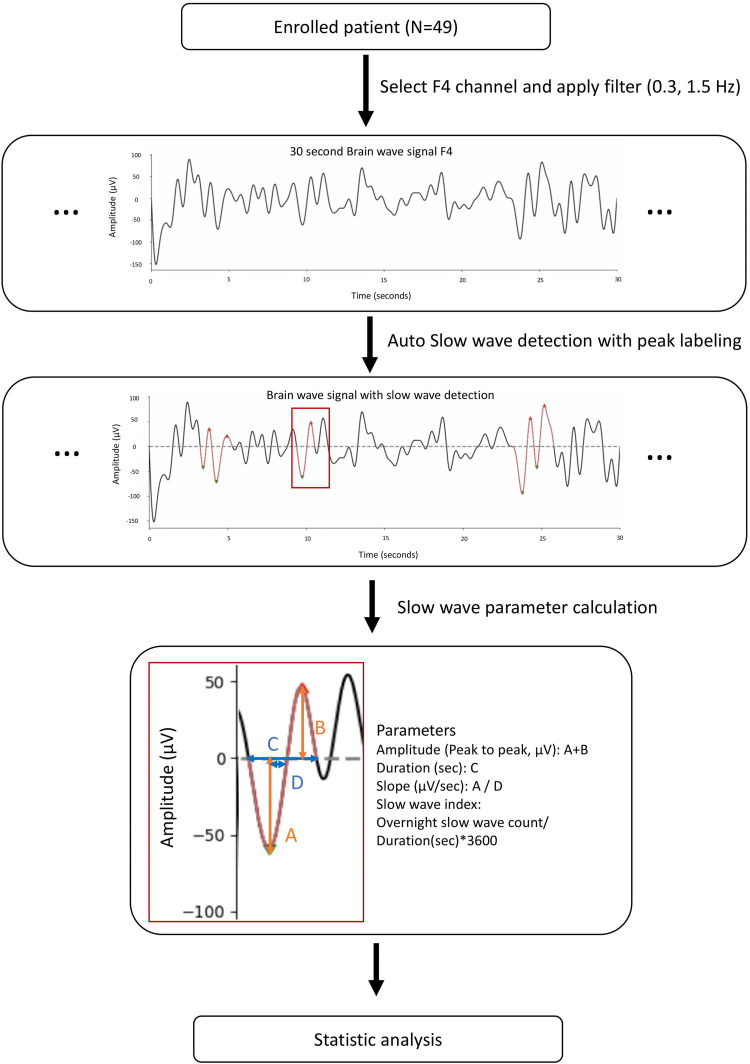
Overview of the slow wave analysis process, including data acquisition, automatic detection, parameter calculation, and statistical analysis.

Preprocessed EEG data were transferred to the Python environment for automated detection of slow waves. Slow waves were detected on the F4 electrode channel continuously throughout the entire night using the YASA open-source Python toolbox (version 0.6.3) (Vallat & Walker, 2021). The detection algorithm followed methods previously described and modified by Massimini et al. (2004) and Carrier et al. (2011). Data were filtered between 0.3 and 1.5 Hz using a finite impulse response (FIR) filter with a transition band of 0.2 Hz to capture slow-wave activity. Slow waves were identified based on several key parameters, including peak-to-peak (PTP) amplitude, negative peak, and positive peak values. As shown in Fig. 1, the PTP amplitude was calculated as the sum of the negative peak (A) and positive peak (B). Negative peaks ranging from -40 to -200 µV and positive peaks ranging from 10 to 150 µV were identified for analysis. Only slow waves with a PTP amplitude exceeding 75 µV were considered significant. The duration of each wave (C) was defined as the time interval between the points where the wave crosses zero before and after both the positive and negative peaks, and the slope (D) was calculated as the amplitude-to-duration ratio. The slow-wave index was calculated as the number of slow waves detected per hour, normalized by the total sleep duration in seconds.

### Cognitive assessment

General cognitive functioning was assessed with the Cognitive Ability Screening Instrument (CASI), a widely used tool for screening cognitive abilities and predicting the risk of dementia. The CASI evaluates multiple domains, including attention, concentration, orientation, short-term and long-term memory, abstraction, judgment, language abilities, visual construction, and list-generating fluency, with a maximum score of 100 (Lin et al., 2012; Tsai et al., 2007). The Word Sequence Learning Test (WSLT) was used to assess both immediate and delayed recall of a sequence of six arbitrary words. Each word consisted of two Chinese characters and was an abstract function word with low semantic imagery, lacking concrete mental representations (Chang et al., 2018). Examinees were asked to repeat the six-word sequence across up to 10 trials during the immediate recall phase. Subsequently, a series of memory tasks—including incidental free recall, cued recall, and recognition tasks—were administered after a 10-minute delay. Nationwide data adjusted for age and education were used to convert performance scores into percentile ranks, including total correct responses, correct position recall, learning across the 10 trials, and the 10-minute free recall, cued recall, and recognition scores. The use of the WSLT as our memory measure was further justified because traditional list-learning tasks typically supra-span items, which can impose greater demands on executive control (Baddeley, 2003). Given that executive dysfunction often persists even among treated OSA patients (Lau et al., 2010; Lau, 2023), we selected the WSLT, which uses sub-span lists and repeated learning trials to reduce executive-related confounding.

A WSLT-based Memory Index Score (WSLT-MIS) was developed for this study, patterned after the Montreal Cognitive Assessment Memory Index Score (MoCA-MIS), which has been widely used to predict the risk of conversion from mild cognitive impairment (MCI) to dementia due to AD (Julayanont et al., 2014). The WSLT-MIS was calculated by summing the number of words recalled in delayed free recall, cued recall, and recognition, each weighted by factors of 3, 2 and 1, respectively, resulting in a total score ranging from 0 to 36. This scoring method was adapted from established clinical practices and mirrors the structure of MoCA-MIS, applying weightings to capture the relative contributions of different recall types. While WSLT-MIS has not been independently validated, it was designed to follow existing neuropsychological conventions.

To evaluate overnight memory efficiency, we calculated both the difference (Δ) value and retention rate, representing the change in WSLT-MIS before and after sleep. The Δ values was computed as: Δ = WSLT-MIS_post-sleep - WSLT-MIS_pre-sleep. The retention rate was calculated to quantify the proportion of retained memory after sleep, relative to pre-sleep performance. The formula aligns with those employed in the Wechsler Memory Scales for assessing the participant’s overall relationship between immediate and delayed memory (Drozdick et al., 2013). To ensure retention rates do not exceed 100 % when post-sleep performance surpasses pre-sleep performance, the denominator takes the maximum of the two values. Specifically, the retention rate ( %) was computed as: $\text{Retention}\;\text{rate}\;\left(\;\%\right)=\frac{\;\text{WSLT}-\text{MIS}\_\text{post}-\text{sleep}}{\text{max}(\text{WSLT}-\text{MIS}\_\text{pre}-\text{sleep},\;\text{WSLT}-\text{MIS}\_\text{post}-\text{sleep})}\times \;100$. A lower retention rate indicates greater forgetting, while a higher retention rate suggests better memory preservation overnight.

### Procedures

**Fig. 2:**
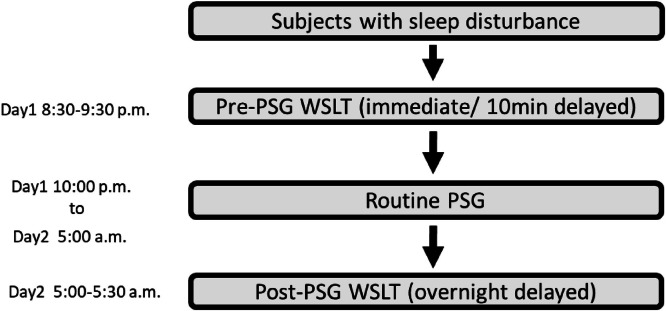
Study flowchart.

Before undergoing PSG, participants were assessed with the WSLT for immediate recall and delayed recall after 10 minutes. Following this assessment, they underwent an overnight PSG examination, during which all physiological signals were collected (Fig. 2). To compare memory performance before and after sleep, PSG data—including sleep stages, sleep architecture, oximetry summary, and slow-wave parameters—were obtained. PSG parameters were first scored by a licensed PSG technician and subsequently reviewed by another technician. Discrepancies in scoring were discussed until a consensus was reached. The scoring technicians were blinded to the cognitive assessment results. After completing the overnight PSG, each participant was asked to recall the words learned from the WSLT the previous night, using free recall, cued-recall, and recognition procedures. Participants identified with elevated AHI were provided with referrals for further evaluation and appropriate clinical management.

### Statistical analysis

This study was designed as a cross-sectional exploratory investigation aimed at identifying potential associations rather than testing predefined hypotheses. Therefore, no a priori sample size calculation was performed. A post-hoc power analysis using G*Power (version 3.1.9.7) was conducted to assess whether the sample size was adequate to detect medium-sized effects.

All statistical analyses were conducted using the Python package “Statsmodels” (version 0.14.0). Within-subject comparisons of WSLT performance before and after sleep were analyzed using paired-sample t-tests. To investigate the associations between sleep parameters and the memory consolidation index, multiple linear regression models were applied. The models were adjusted for age, body mass index (BMI), and years of education to control for potential confounding factors. Statistical results were reported with both unstandardized and standardized coefficients, along with 95 % confidence intervals (CIs). Significance was determined at *p* < 0.05.

## Results

### Participants and exclusions

Initially, 59 participants were recruited; however, 10 were excluded due to technical issues affecting PSG data integrity (n =5), concurrent antidepressant use (n =2), and absence of REM sleep during recording (n = 3).

### Demographics and baseline characteristics

The baseline characteristics of the 49 participants included in this study (23 males and 26 females) are presented in Table 1. The mean age was 59.51 ± 11.17 years (range: 48 –71 years). The mean BMI was 24.20 ± 3.38 kg/m^2^, with a mean neck circumference of 35.81 ± 5.67 cm and mean waist circumference of 85.41 ± 11.00 cm. The participants were categorized by OSA: 11 (22 %) were non-OSA, 12 (24 %) had mild OSA, 19 (39 %) had moderate OSA, and 7 (14 %) had severe OSA. Cognitive screening using the CASI showed a mean score of 93.73 ± 4.51. Performances on the WSLT prior to overnight PSG were slightly below the normative reference values (based on age- and education-matched norms), with Z-scores as follows: Immediate Recall (-0.10 ± 1.06), Delayed Recall (-0.42 ± 1.27), Cued Recall (-0.07 ± 1.23), and Recognition score (-0.28 ± 1.09).

**Table 1:** Demographic characteristics of the participants (N = 49).

Categorical Variables	Value
Age (years)	59.51 ± 11.17
Gender (Male)	23 (47 %)
Education (years)	12.78 ± 3.32
BMI (kg/m^2^)	24.20 ± 3.38
Neck circumference (cm)	35.81 ± 5.67
Waist circumference (cm)	85.41 ± 11.00
**OSA severity, N (%)**	
Normal	11 (22%)
Mild	12 (24 %)
Moderate	19 (39 %)
Severe	7 (14 %)
**Cognitive function**	
CASI score	93.73 ± 4.51
WSLT score (Z-score)	
Immediate Recall	-0.10 ± 1.06
Delayed Recall	-0.42 ± 1.27
Cued Recall	-0.07 ± 1.23
Recognition	-0.28 ± 1.09

Abbreviations: BMI: Body mass index; CASI: Cognitive Abilities Screening Instrument; OSA: Obstructive sleep apnea; WSLT: Word Sequence Learning Test.

Data are denoted by mean ± standard deviation or numbers (percentage).

### Overnight changes in cognition function

Table 2 presents the overnight changes in memory function as measured by the WSLT. The changes observed in the various WSLT recall types were as follows: free delayed recall: -0.18 ± 1.63, cued recall: -0.55 ± 1.57, and recognition: -0.29 ± 1.43. Paired-sample t-tests indicated that both cued recall and recognition performance declined significantly after sleep compared to baseline (p < 0.05). Post-sleep cued-recall and recognition performances showed a significant decline compared to pre-sleep values (p < 0.05). The memory consolidation index showed a reduction in WSLT-MIS of -1.94 ± 7.89, with a retention rate of 81.23 ± 23.68 %.

**Table 2:** Overnight changes in WSLT scores assessed by paired-sample t tests (N = 49).

Categorical Variables	Value
**Δ WSLT score**	
Δ Recall	-0.18 ± 1.63
Δ Cued	-0.55 ± 1.57*
Δ Recognition	-0.29 ± 1.43^⁎⁎^
**WSLT-MIS**	
Δ Value	-1.94 ± 7.89
Retention rate ( %)	81.23 ± 23.68

Abbreviations: WSLT: Word Sequence Learning Test; MIS: Memory Index Score.

Note: Difference value (Δ) was calculated by subtracting post-sleep score from pre-sleep score.

Data are denoted by mean ± standard deviation.

### Sleep architecture, sleep disorder indexes, and slow wave characteristics

For sleep disorder indexes (Table 3), participants exhibited an ODI-3 % of 13.84 ± 13.99 events/hour, an AHI of 17.75 ± 14.45 events/hour, and an ArI of 13.79 ± 5.79 events/hour. The slow wave sleep features included a mean PTP amplitude of 117.32 ± 11.95 µV, a mean slow wave slope of 393.94 ± 66.68 µV/sec, and a mean slow wave duration of 1.33 ± 0.13 seconds. The slow wave index was 61.54 ± 68.92 events/hour.

**Table 3:** Sleep architecture, sleep disorder indexes, and slow wave characteristics of the participants (N = 49).

Categorical Variables	
**Sleep architecture**	
Sleep efficiency ( %)	71.47 ± 15.86
Wake ( % of SPT)	21.86 ± 15.64
NREM ( % of SPT)	65.93 ± 13.22
REM ( % of SPT)	12.07 ± 6.31
**Sleep disorder index** (events/h)	
ODI-3 %	13.84 ± 13.99
AHI AHI in NREM AHI in REM	17.75 ± 14.45 15.17 ± 14.44 33.35 ± 23.74
ArI ArI in NREM ArI in REM	13.79 ± 5.79 13.18 ± 6.04 18.00 ± 12.22
**Slow wave characteristics**	
Mean of PTP (µV)	117.32 ± 11.95
Mean of slope (µV/sec)	393.94 ± 66.68
Mean of duration (sec)	1.33 ± 0.13
Slow wave index (events/h)	61.54 ± 68.92

Abbreviations: AHI: Apnea-hypopnea index; ArI: Arousal index; NREM: Non-rapid eye movement; ODI-3 %: Oxygen desaturation index ≥3 %; PTP: Peak-to-peak amplitude; REM: Rapid eye movement; Slope: Slope between negative peak and mid-crossing; SPT: Sleep period of time.

Data are denoted by mean ± standard deviation.

### Association between sleep parameters and memory consolidation measures

Multivariate regression results examining both AHI and slow-wave index as independent predictors of memory performance are presented in Table 4, Table 5. These analyses demonstrate that both variables contributed uniquely to WSLT-MIS scores after controlling for age, BMI, and education. As presented in Table 4, the associations between various sleep parameters and the change in WSLT-MIS for the 49 patients were analyzed using adjusted linear regression models accounting for age, BMI, and education. In the adjusted model, ODI-3 % was significantly negatively associated with the difference score in WSLT-MIS (B = -0.24, β = -0.42, 95 % CI: -0.77 to -0.07, p < 0.05), indicating that an increase of approximately 4.17 desaturation events per hour correspond to a 1-point reduction in ΔWSLT-MIS. Similarly, AHI was significantly negatively associated with WSLT-MIS changes, particularly during non-REM (NREM) sleep (B = -0.20, β = -0.36, 95 % CI: -0.70 to -0.03, p < 0.05), while no significant associations were observed for AHI during REM sleep. This suggests that an increase of 5 apnea-hypopnea events per hour during NREM sleep is associated with a 1-point decrease in Δ WSLT-MIS. In contrast, the slow-wave index was positively associated with changes in WSLT-MIS (B = 0.31, β = 0.36, 95 % CI: 0.08 to 0.63, p < 0.05), indicating that an increase of approximately 3.23 slow-wave events per hour corresponded to a 1-point improvement in ΔWSLT-MIS. Other parameters, including ArI and additional slow-wave characteristics, were not significantly associated with WSLT-MIS changes.

**Table 4:** Associations between sleep parameters and the difference in WSLT-MIS (N = 49).

Categorical Variables	Regression coefficient ^a^ (95 % confidence interval)	
	**Unstandardized (B)**	**Standardized (β)**
**Sleep disorder index** (events/h)		
ODI-3 %	-0.24 (-0.44 to -0.03)*	-0.42 (-0.77 to -0.07)*
AHI	-0.19 (-0.38 to 0.01)	-0.34 (-0.69 to 0.01)
AHI in NREM	-0.20 (-0.39 to 0.01)*	-0.36 (-0.70 to -0.03)*
AHI in REM	0.04 (-0.07 to 0.15)	0.12 (-0.20 to 0.44)
ArI	-0.10 (-0.49 to 0.29)	-0.08 (-0.35 to 0.20)
ArI in NREM	-0.10 (-0.47 to 0.28)	-0.08 (-0.35 to 0.20)
ArI in REM	0.05 (-0.13 to 0.24)	0.08 (-0.20 to 0.36)
**Slow wave characteristics**		
Mean of PTP (µV)	0.07 (-0.12 to 0.26)	0.11 (-0.18 to 0.39)
Mean of Slope (µV/sec)	0.14 (-0.15 to 0.42)	0.18 (-0.10 to 0.46)
Mean of Duration (sec)	-0.09 (-0.37 to 0.20)	-0.15 (-0.45 to 0.15)
Slow wave index (events/h)	0.31 (0.03 to 0.58)*	0.36 (0.08 to 0.63)*

Abbreviations: AHI: Apnea-hypopnea index; ArI: Arousal index; NREM: Non-rapid eye movement; ODI-3 %: Oxygen desaturation index ≥3 %; PTP: Peak-to-peak amplitude; REM: Rapid eye movement; Slope: Slope between negative peak and mid-crossing; WSLT-MIS: Word Sequence Learning Test-Memory Index Score.

Note: ^a^ Results are from multivariate linear regression models adjusted for age, body mass index, and years of education.

⁎ *p* < 0.05

**Table 5:** Associations between sleep parameters and the retention rate of the WSLT-MIS (N = 49).

Categorical Variables	Regression coefficient ^a^ (95 % confidence interval)	
	**Unstandardized (B)**	**Standardized (β)**
**Sleep disorder index** (events/h)		
ODI-3 %	-0.50 (-1.11 to 0.17)	-0.29 (-0.65 to 0.06)
AHI	-0.42 (-1.01 to 0.18)	-0.25 (-0.60 to 0.10)
AHI in NREM	-0.46 (-1.03 to 0.10)	-0.28 (-0.62 to 0.05)
AHI in REM	0.03 (-0.25 to 0.32)	0.09 (-0.23 to 0.41)
ArI	-0.53 (-1.67 to 0.61)	-0.13 (-0.40 to 0.14)
ArI in NREM	-0.63 (-1.72 to 0.46)	-0.16 (-0.43 to 0.11)
ArI in REM	0.23 (-0.31 to 0.77)	0.12 (-0.15 to 0.39)
**Slow wave characteristics**		
Mean of PTP (µV)	0.15 (-0.42 to 0.72)	0.07 (-0.20 to 0.35)
Mean of Slope (µV/sec)	0.05 (-0.06 to 0.15)	0.13 (-0.15 to 0.41)
Mean of Duration (sec)	-21.87 (-76.27 to 32.53)	-0.12 (-0.42 to 0.17)
Slow wave index (events/h)	0.14 (0.05 to 0.23)^⁎⁎^	0.40 (0.15 to 0.66)^⁎⁎^

Abbreviations: AHI: Apnea-hypopnea index; ArI: Arousal index; NREM: Non-rapid eye movement; ODI-3 %: Oxygen desaturation index ≥3 %; PTP: Peak-to-peak amplitude; REM: Rapid eye movement; Slope: Slope between negative peak and mid-crossing; WSLT-MIS: Word Sequence Learning Test-Memory Index Score.

Note: ^a^ Results are from multivariate linear regression models adjusted for age, body mass index, and years of education. ^⁎^*p* < 0.05, ^⁎⁎^*p* < 0.01.

Table 5 presents the associations between various sleep parameters and the retention rate of WSLT-MIS, analyzed using the same adjusted regression models. The slow-wave index was significantly positively associated with the WSLT-MIS retention rate (B = 0.14, β = 0.40, 95 % CI: 0.15 to 0.66, p < 0.01), indicating that each additional slow-wave event per hour corresponded to a 0.14-point increase in the retention rate. This association was both strong and statistically significant effect. In contrast, no other sleep parameters, including ODI-3 %, AHI, ArI, or other slow-wave characteristics, were significantly associated with the retention rate.

Based on the lowest statistically significant standardized beta coefficient (β = 0.36) observed in Table 4, Table 5, a medium effect size of f² = 0.15 was assumed for post-hoc power analysis. With an alpha level of 0.05 and four predictors (three covariates and one primary predictor), the calculated statistical power was approximately 0.845 (84.5 %). This suggests that the study was sufficiently powered to detect medium-sized effects in the multivariate regression analyses.

## Discussion

This study investigated the relationship between sleep parameters, specifically the characteristics of SWS, and their impact on memory consolidation in individuals with OSA. Our findings highlight the significant role of nocturnal oxygen desaturation and slow-wave activity in the memory consolidation process.

Consistent with previous research, our study revealed that both AHI and ODI-3 % were significantly associated with declines in memory consolidation indices. These findings align with studies demonstrating the adverse effects of OSA on cognitive functions, particularly memory (Bucks et al., 2013; Marchi et al., 2023). The association between nocturnal oxygen desaturation and impaired memory performance is likely due to intermittent hypoxia and sleep fragmentation, both of which disrupt memory consolidation (Fernandes et al., 2022). For instance, studies have shown a significant reduction in post-sleep verbal retention rates among patients with OSA compared to healthy individuals (Kloepfer et al., 2009). Moreover, patients who underwent Continuous Positive Airway Pressure (CPAP) treatment for three months exhibited improved overnight declarative memory performance compared to those who did not receive treatment (Djonlagic et al., 2021). CPAP is thought to extend SWS duration, enhance the power and amplitude of slow waves during NREM sleep (Li et al., 2023), and reduce neurochemical biomarkers associated with neurodegenerative diseases (Liu et al., 2023). Furthermore, the present study found that AHI during NREM sleep had a stronger impact on memory consolidation compared to AHI during REM sleep, supporting the hypothesis that declarative memory formation is closely linked to NREM sleep (Gais & Born, 2004).

Additionally, our findings indicate that the slow-wave index was significantly associated with memory consolidation efficiency, even after adjusting for age, BMI, and education level. This result is consistent with previous studies emphasizing the critical role of SWS in declarative memory consolidation (Gais & Born, 2004; Walker, 2009). Slow waves are believed to facilitate the reactivation and integration of newly acquired memories within hippocampal and neocortical networks (Miyamoto et al., 2017; Takashima et al., 2006). Disruptions in slow-wave activity have been linked to poorer performance in episodic memory tasks, such as word-pair tests (Mander et al., 2013). Moreover, studies have shown that artificially enhancing slow oscillations during sleep can improve declarative memory performance, further underscoring the importance of slow waves in memory processes (Marshall et al., 2006; Mölle & Born, 2011).

A notable implication of our study is that some participants with normal performance on general cognitive screens may still exhibit subtle deficits in memory consolidation (Georgeson et al., 2025). This underscores the value of incorporating domain-specific assessments, particularly for memory, when evaluating individuals with sleep-related complaints or suspected OSA (Wallace & Bucks, 2013). General cognitive screening tools often fail to detect subtle or domain-specific impairments (Cullen et al., 2007), which is especially important in light of memory’s vulnerability to sleep-related disruptions. Although our within-subject design was intended to isolate sleep-related changes in memory consolidation while minimizing inter-individual differences in baseline attention or executive function (Lau, 2023), we acknowledge that memory is a multifaceted construct. The use of a single verbal memory task may limit the generalizability of our findings. Future research should incorporate a broader cognitive battery to better characterize the domain-specific cognitive effects of sleep architecture and sleep-disordered breathing.

This study applied previously validated slow-wave detection algorithms to analyze EEG recordings, providing an automated and objective alternative to manual PSG scoring, which is prone to human error and inter-rater variability. By applying these algorithms, we obtained more consistent and standardized data interpretation, reducing the subjectivity associated with traditional manual scoring. Automation significantly reduces the time required to process large datasets, making it more efficient and scalable compared to the labor-intensive nature of manual PSG scoring (Zhang et al., 2024). Additionally, these algorithms quantify key EEG features, including slow-wave frequency, amplitude, and density, offering deeper insights into sleep quality beyond conventional methods that primarily categorize sleep stages. By standardizing sleep analysis, they minimize observer bias and enhance result reproducibility across studies.

This study has several strengths. The use of overnight PSG provided accurate and objective measurements of sleep parameters such as AHI and ODI, complemented by validated cognitive assessments, allowing for a comprehensive evaluation of the impact of OSA on memory consolidation. Additionally, the study utilized existing automated algorithms for SWS detection, enabling precise quantification of slow-wave activity while reducing observer bias and improving the reliability of EEG-based analyses. However, certain limitations should be acknowledged. First, the relatively small sample size may limit the generalizability of the findings. Second, although we controlled for educational level and excluded participants with major neuropsychiatric disorders, as well as later excluded two participants due to antidepressant use, other potential confounders, such as vascular disease, diabetes, chronic obstructive pulmonary disease, and chronic kidney disease, were not systematically accounted for. While their direct impact on memory consolidation remains unclear, their associations with sleep disturbances warrant consideration in future studies. Moreover, while structured cognitive tests effectively assessed memory consolidation, memory processing is complex. Advanced neuroimaging techniques, such as functional MRI, and biomarkers could provide deeper insights into underlying neural mechanisms. Finally, the study’s cross-sectional design limits causal inferences. Future longitudinal studies are needed to explore the temporal relationships between sleep disturbances and cognitive decline, as well as the potential benefits of targeted sleep Memory Index Score on memory function.

## Conclusion

In conclusion, this study demonstrates that slow-wave activity plays a critical role in memory consolidation during sleep. The associations between reduced oxygen saturation, increased AHI, and impaired memory performance underscore the detrimental effects of sleep disruptions on cognitive function, particularly in individuals with OSA. These findings suggest that targeting slow-wave sleep may be beneficial in therapeutic interventions. While CPAP therapy remains the primary treatment for improving oxygenation and reducing sleep fragmentation, our findings suggest that enhancing SWS itself may further support memory consolidation. Similarly, increasing SWS may enhance memory function in other sleep disorders, such as insomnia and narcolepsy. Future research would benefit from exploring these relationships using functional MRI and EEG-based connectivity analyses, along with longitudinal designs, to better establish causal links and assess the long-term impact of SWS-enhancing interventions on cognitive health.

## Declaration of competing interest

The authors declare that they have no known competing financial interests or personal relationships that could have appeared to influence the work reported in this paper.
